# Medicine in America

**Published:** 1918-08-17

**Authors:** W. Osler

**Affiliations:** Regius Professor of Medicine, Oxford University.


### MEDICINE IN AMERICA.

**SPEECH BY SIR W. OSLER, Bart., F.R.S., Regius Professor of Medicine, Oxford University.**

Speaking to University Extension students at their summer meeting at Cambridge, Sir William Osler, Regius Professor at Oxford, described the growth of medicine in the United States, claiming that up to the present war-time the three great contributions of the New World had been quinine, the use of surgical anæsthesia, and bacteriological discoveries. In the early colonial days eminent doctors had been few in the Colonies. Hawthorne's picture of Roger Chillingworth in "The Scarlet Letter" might be trusted to be true to life. One Fuller was both doctor and deacon on the *Mayflower*. Most were graduates of Cambridge or Leyden, and were surgeons or barber-surgeons. They had to face hard conditions. Dr. Dinley was lost in a blizzard while returning from a case, and his little son, born ten days later, received the name of "Fathergone." They lived partly by other vocations; one was a schoolmaster, another a butcher, one kept a tavern, there was one "woman practitioner." Presently colonists began to send a son to study at Edinburgh or Leyden. Some superstition had to be cleared away. Cotton Mather declared all disease to be the scourge of God for the sins of men. In 1765 John Morgan, a pupil of John Hunter, wrote a discourse on medical schools, five of which had actually been founded before 1500. The first hospitals were to be found in French colonies, as at Quebec; the first in the States was at Philadelphia, and help was given to it by Benjamin Franklin, who himself dabbled in medicine, being consulted by letter frequently by ladies. He also influenced the introduction of electricity as treatment for disease. Mental suffering was studied; John Rush, a contemporary of Morgan, wrote a treatise on Insanity. James Jackson, 1777-1848 was author of "Letters to a Young Physician"; his descendants are doctors in New England States to-day.

Ephraim McDowell, born 1771, working in the Kentucky village of Danville, far out in the prairie, was first to operate for ovarian cyst, and records eight successes out of thirteen operations. Daniel Drake (1785-1852) studied diseases along the course of the Mississippi. Some twenty-five of the best American doctors of the nineteenth century owed their skill to the teaching in Paris of François Brousset (1772-1830); of René Laennec (1781-1825), introducer of the stethoscope; of Pierre Louis, the earnest student of statistics who, by his indications of results, ousted the practice of bleeding. François Majendie was a distinguished pupil. But on October 16, 1846, a great event took place. The credit of an invention belongs more to the man who gives it to the world than to the actual discoverer, and on this date Dr. Morton, a dentist, removed a small facial tumour from a patient in the Massachusetts General Hospital with concurrence of the doctors. The lecturer showed on the screen a reproduction of an old print showing the scene. Hemlock and alcohol were finally superseded, and in three months the new anæsthesia was being used everywhere. From 1860 to 1890 American medicine came largely under German influences, especially those of specialists in eye and skin diseases. Skodé in Vienna, the admirer of England, was stemming the flow of promiscuous drug-giving. Rudolph Virchow, editor, archæologist, politician, and accomplished medical scientist, was teaching. Koch (1843-1910) contributed a great impetus to American study of bacteriology, and Ludwig Traube to clinical physiology—the use of the laboratory grew. The third period shows Americans studying at home. Private adventure medical schools multiplied, even in connection with Universities. In 1874 MacGill University had a medical school receiving State grant, but completely uncontrolled. Abuses naturally arose. At Harvard the University President, Elliott, surprised the professors by suddenly appearing and occupying the chair. Dr. Pepper helped in reforms and presently not only schools but attached hospitals became recognised parts of Universities. At Johns Hopkins to-day some forty to fifty men work in the laboratory, and there is a clinic all under control of Dr. Halstead.

A wonderful feature in America is the famous clinic of the brothers Mayo in the little State of Minnesota, a small prairie town of Rochester, Their dominance of environment has brought many men to take a last journey for learning's sake. The era of preventive medicine dawned when the yellow fever, raging in Cuba especially, but travelling north even to Philadelphia and Baltimore, was traced by Walter Reed to the mosquito. Courageous volunteers slept in beds vacated by dead victims, but a mosquito-bitten investigator died. The oiling and draining of swamps proved a cure, and after 150 years of terror the disease was practically wiped out of Cuba and Central America. This, said the Regius Professor, made multi-millionaires "sit up." Here was indeed a use for vast wealth. The Carnegie Trust published Abraham Flexner's Report on Medical Education, which was the death of some worthless medical schools, and other reports, of which some closely concern us in England, and are the outcome of vast and minute study. The Rockefeller Institute studied such matters as hookworm diseases in the tropics and malaria in Arkansas, where 600 doctors' calls in 1914 were reduced to forty-six in 1915 and further to fourteen. The schools of study established by this trust at Pekin and elsewhere in China promise an immense future for bacteriology. The value of the great journals of medical science can hardly, in Sir W. Osler's view, be overrated as teaching the daily discoveries. The doctors in America whose copies of the *American Medical Journal* or *Journal of American Medical Association* are uncut do not deserve a second visit. Listeners may have thought again of the spirit shown by that eminent doctor who said at the recent conference in London, "*We Americans are here as learners.*" It is a spirit to be maintained among ourselves also.

